# Quality of Life and Activities of Daily Living among Patients with Complete Cervical Spinal Cord Injury and Surgical Treatment in Vietnam

**DOI:** 10.3390/ijerph18189703

**Published:** 2021-09-15

**Authors:** Nguyen Le Bao Tien, Vo Van Thanh, Khuc Thi Hong Hanh, Pham Gia Anh, Le Thi Minh Huyen, Ngo Thanh Tu, Dang Thi Ngoc Mai, Phung Lam Toi

**Affiliations:** 1 Institute of Orthopaedics and Trauma Surgery, Viet Duc Hospital, Hanoi 100000, Vietnam; DrNguyenlebaotien@vduh.org (N.L.B.T.); thanhvo@hmu.edu.vn (V.V.T.); Bacsingothanhtu@vduh.org (N.T.T.); 2 Department of Surgery, Hanoi Medical University, Hanoi 100000, Vietnam; 3 Institute for Preventive Medicine and Public Health, Hanoi Medical University, Hanoi 100000, Vietnam; 1553020025@daihocyhanoi.edu.vn (K.T.H.H.); 1553020040@daihocyhanoi.edu.vn (L.T.M.H.); 4 Oncology Department, Viet Duc Hospital, Hanoi 100000, Vietnam; Phamgiaanh@vduh.org; 5 Center of clinical pharmacology, Hanoi Medical University, Hanoi 100000, Vietnam; Dangngocmai@hmu.edu.vn; 6 Health Strategy and Policy Institute, Ministry of Health, Hanoi 100000, Vietnam

**Keywords:** quality of life, spinal cord injury, surgery, ADL, Vietnam

## Abstract

Spinal cord injury (SCI) is defined as temporary or permanent changes in spinal cord function and reflex activity. The objective of this study is to evaluate health-related quality of life (HRQoL) and activities of daily living (ADL) among postoperative surgery patients with complete cervical SCI in Vietnam and to explore the factors associated with these indices. A cross-sectional study was conducted on 88 adults in Vietnam from June 2018 to June 2019. The EQ-5D-5L, ADL, and instrumental activities of daily living (IADL) were applied. Multivariate Tobit regression was adopted to determine factors that were associated with HRQOL, ADL, and IADL. Results: Participants who were in American Spinal Cord Injury Association (ASIA) scale group A (ASIA-A) had the lowest ADL, IADL index, and HRQOL score (*p* < 0.001). HRQoL and ADL were affected by health insurance coverage, occupation, type of fracture, and IADL. Meanwhile, IADL was significantly associated with living areas and ASIA. Low HRQoL among patients suffering from SCI was observed. Attention should be given to outcomes related to a disability during clinical treatment and should be treated effectively in the recovery.

## 1. Introduction

Spinal cord injury (SCI) is a severe condition that results in several consequences such as autonomic nervous system disturbances, increased risk for upper urinary tract infections due to loss of bladder and bowel control, and sexual disfunction—all together affecting the quality of life of patients [1]. In previous studies, around 39% to 41.4% of SCI were attributed to complete SCI that causes an irreversible loss of function below the site of lesion and leads to permanent disability [2,3,4]. Moreover, complete cervical SCI has the biggest share among complete SCI [3]. 

In 2016, approximately 27.04 million (24.97–30.14 million) new cases of SCI were recorded [5]. The highest incidence rates of SCI were in high-income North America, Western Europe, and the high-income Asia Pacific. The incidence of SCI ranged from 10.6 to 22.6 per million in Québec, Canada (2000–2011) [6]. In Finland, it was reported that the incidence of SCI was between 25.1 and 38.1 per million (2012–2013) [7]. Moreover, the incidence of SCI is higher in developing countries compared with that in developed countries with the same trends for mortality rate. From 2004 to 2008, the estimated annual incidence of SCI in China was 23.7 per million [8]. Evidence shows that developing countries have the highest 1-year mortality rates [9,10]. Besides, Asian countries lack the appropriate epidemiologic data on SCI [11]. In Vietnam, for example, an estimation in 2007 showed that the prevalence and incidence of SCI were 464 and 13.9 per million [12]. The majority of these patients have a high rate of experiencing all health problems with mobility, self-care, usual activity, pain, and anxiety [13]. Furthermore, the average costs of an injury, including direct medical, direct non-medical, and indirect costs, were at USD 363, more than 6 months of the average salary in Vietnam [14].

SCI patients are facing various health problems. SCI leads to impairment of the respiratory system as shortness of breath, and SCI patients with dyspnea report lower general health, mental health, and vitality [15]. Furthermore, patients with injury at the cervical level report lower quality of life than those whose injuries are beyond the cervical level [16]. Among male patients, SCI is also associated with sexual impairments, which in turn affect the quality of life [17]. In addition to health-related quality of life (HRQoL), disability is also a relevant outcome for SCI patients. Functional impairments due to SCI are highly variable and may impact a person’s capacity to engage in activities of daily living (ADL) and social participation. In previous studies, the performance of daily activities which included basic and complex functions (ADLs and IADLs, respectively) were also assessed. Activities of daily living (ADLs), often termed physical ADLs or basic ADLs, include the fundamental skills typically needed to manage basic physical needs, comprising the following areas: grooming/personal hygiene, dressing, toileting/continence, transferring/ambulating, and eating. Basic ADLs are generally categorized separately from Instrumental Activities of Daily Living (IADLs), which include more complex activities related to independent living in the community (e.g., managing finances and medications). IADL is more varied and typically performed in a greater range of settings than ADL. Besides, direct assessment of ADL and IADL appear to be the best predictor of an individual’s need for support or ability to function independently in the community [18]. Individuals with higher levels of injury may require complete assistance with ADLs, which has the potential to impact social participation, whereas those with lower or incomplete injuries may experience more independence in all areas 2000 [19]. Through ongoing assistance with ADLs and IADLs for individuals with SCI, caregivers can improve access to the community and increase social participation, including employment and adapted sports and recreation opportunities 1998 [20]. 

Understanding the ability of SCI patients to perform daily activities is also crucial to evaluate treatment outcomes as well as monitor for long-term care. While the evidence was predominantly from the developed world [6,7], evidence is still not well documented in terms of quality of life and ADL among postoperative surgery patients with complete cervical spinal cord injury in Vietnam. This study aimed to investigate the HRQoL, ADL among complete spinal cord injury patients in Vietnam. Information regarding HRQoL and activities of daily living among SCI patients in a developing country such as Vietnam is beneficial to global knowledge as well as relevant stakeholders. Besides, the aim was for medical care and follow-up planning as well as for preventive initiatives. However, as far as we know, well-designed studies of SCI in Vietnam populations are scarce. This could provide useful insight for health practitioners in the country regarding the patient’s outcome of current treatment therapy and consequently enable the comparison to future potential treatments.

## 2. Materials and Methods

### 2.1. Setting and Sampling Method

A cross-sectional study was conducted in Viet Duc Hospital—a central hospital specialized in surgery in Hanoi, Vietnam from June 2018 to June 2019. 

Patients were selected by convenience sampling approach based on the list of patients with cervical SCI and undergone surgery at the hospital. Patients were invited to participate in this study at the time he/she came back for a checkup. All patients met the following eligibility criteria: (1) diagnosed with complete cervical spinal cord injury and received surgery treatment, (2) with nerve damage (i.e., AIS from A to D), (3) with normal verbal communication; (4) willing to participate in the survey were contacted again to re-examination and invited to participate in the research. We excluded patients having multiple traumatic injuries, having comorbidity such as diabetes or cancer, as these factors may significantly influence the quality of life and do not reflect the actual situation of SCI patients. Most of the invited patients were accepted to part take in the survey, no one appeared to be cognitively compromised; 12 patients who had comorbidities (i.e., diabetes or hypertension) were excluded, six patients were lost to follow-up. Accordingly, a total of 88 patients were included in the study.

Before the interview, patients were introduced to the purpose of the study, risks, and benefits of participating in the survey in the written informed consent form. Moreover, participants’ information related to clinical characteristics such as level of injury and AIS score was obtained from medical records. Data collection was completed by medical doctors who were trained by methodologists and epidemiologists to ensure the quality of data. Data collection was also piloted in a small group of patients (10 patients) to finetune the questionnaires. 

### 2.2. Measurement and Instruments

#### 2.2.1. Demographic Characteristics

The following information was collected from patients’ interviews: age, gender, level of education, marital status, occupation before and after having SCI, living area, health insurance coverage status. 

#### 2.2.2. Injury-Related Characteristics

Participants reported their cause of injuries such as traffic or occupational accident. Additionally, information on the type of fracture such as burst cervical vertebrae and/or compression, ASIA was retrieved from the medical record. ASIA investigation was done by a medical doctor during the examination and classified into four groups: A (complete disfunction), B (sensory incomplete), C (>50% motor incomplete), D (≤50% motor incomplete) based on the level of motor and sensor dysfunction [21].

#### 2.2.3. Health-Related Quality of Life (HRQoL)

We examined the HRQoL by using the EuroQol-5 dimensions-5 levels (EQ-5D-5L). The EQ-5D-5L answers were then converted into utility based on the Vietnamese value set. An EQ-5D-5L value set based on the Vietnamese adult population was effectively developed by using both composite time trade-off and discrete choice experiment methods. The instrument was converted to utility scores by using the Vietnamese value set [22]. In this study, five dimensions were assessed including mobility, self-care, usual activities, pain/discomfort, and anxiety/depression. A Likert scale with five response levels from no problem to extreme problem was utilized to rate each dimension. 

#### 2.2.4. Activities of Daily Living

We assessed the activities of daily living (ADL) by applying the Katz ADL index [23] questionnaire. A list of activities was evaluated to assess fundamental skills that are required to independently care for oneself such as eating, bathing, and mobility. Besides, the instrumental activities of daily living (IADL) were also assessed by the IADL scale developed by Lawton and Brody [24]. The IADL scores relate to tasks that require sufficient capacity to make decisions as well as greater interaction with the environment [25]. IADLs facilitate independent living and include telephone use, food preparation, medication management, driving or traveling, financial management, meal preparation, housekeeping, laundry, and shopping [26]. Both the Katz ADL index and IADL scale have been validated and applied in the Vietnamese setting previously [27,28]. 

### 2.3. Statistical Analysis

Variables were presented through descriptive statistics (i.e., frequency, percentage, mean, and standard deviation (SD)). The Mann–Whitney U test and Kruskal–Wallis test was performed to test the statistical significance of HRQoL, ADL, and IADL between two and more than two, respectively categories of the variable. We applied multivariate Tobit regression models for identifying associated factors with HRQoL, ADL, and IADL score because there was either left- or right-censoring in the indices (dependent variable). Specifically, utility (HRQoL value) had the upper limit was 1, ADL score ranged from 0 to 6, and IADL scores ranged from 0 to 8. Independent variables including demographic characteristics, cause of injuries, type of injuries, and ASIA, which had *p*-value < 0.1 in the univariate regression models between them and outcome indices were selected in the Tobit regression model. These models were accompanied by a stepwise backward selection strategy. The threshold of *p* < 0.1 for the log-likelihood ratio test was used to produce reduced models. A *p*-value of 0.05 was applied as the threshold of statistical significance. All data analysis was performed in STATA 15.0 (Stata Corp. LP, College Station, TX, USA). 

### 2.4. Ethical Approval

The study protocol was assessed and approved by the Director Broad of Viet Duc Hospital (Code: 15.2018.NCVD). The patient’s information was kept confidential and only used for research.

## 3. Results

### 3.1. Patients’ Characteristics

A total of 88 patients participated in this study with a mean age of 47.7 years (SD = 14.6). Among them, 87.5% were male, half of them had high school education or above, most of them (89.8%) got married, 48.9% were unemployed, 82.9% lived in rural areas, and 79.6% had health insurance. Traffic accidents were the most common cause of injuries, accounted for 51.1% of the patients. Regarding the type of injuries, 31.8% of patients were compression fracture while 30.7% were burst. The proportion of ASIA group D and C were 28.4% and 25.0%, respectively. The average length of stay in the hospital was 12.91 ± 5.86. Time from surgery to interview was 18.5 ± 5.2 months (Table 1).

### 3.2. Health-Related Quality of Life, ADL, and IADL

All the three indices (HRQOL, ADL, and IADL) were remarkably influenced by the occupation. Students had highest quality of life indices both before the accident (HRQOL = 0.97, (*p* < 0.05), ADL = 6.00, (*p* < 0.05), IADL = 8.00) and after the accident (HRQOL = 0.97, ADL = 6.00, IADL = 8.00, *p* < 0.001) (Table 2). Whereas, unemployed participants were reported to have the lowest points in the HRQOL index before and after the unfortunate accidents (which are 0.22 and 0.30, *p* < 0.001). Similar findings were found in ADL and IADL indices with the lowest scores in a group of unemployed patients. We also found participants living in urban areas (0.63) had higher mean HRQOL than those living in the countryside (0.47, *p* < 0.05). Meanwhile, no statistically significant difference in ADL and IADL scores was found regarding the living area. 

In Table 2, the results showed that all the three indices (HRQOL, ADL, and IADL) were remarkably influenced by ASIA. ASIA-D had highest scores in the HRQOL, ADL and IADL index (HRQOL = 0.79, ADL = 5.60, IADL = 7.60, *p* < 0.001). We also found that having compression fracture injuries was significantly associated with the HRQOL.

Overall, no statistical difference in HRQOL, ADL, and IADL scores was found regarding age, gender, health insurance coverage, educational level, marital status, and cause of injuries. Patients who were under 30-year-old or without health insurance and living alone had the highest mean HRQoL scores (0.65, 0.59, 0.59, respectively) and ADL scores (4.91, 4.33, 4.33, respectively). Males had higher scores in both indices than females in all three indices. Additionally, the group of participants covered by health insurance and people living with a partner had a higher score for the ADL index but lower IADL scores than the other groups (Table 2).

### 3.3. Factors Associated with HRQOL, ADL, and IADL

Older respondents, those who lived with husband/wife, and those who had higher IADL scores were more likely to have better HRQoL. However, insured people, those who retired, traffic accidents, and in group A of ASIA were significantly correlated with lower HRQoL (Table 3).

For ADL, females, insured patients, retirement/unemployment had significantly lower ADL scores. Meanwhile, patients who had burst or compression fracture injuries and higher IADL scores were more likely to have higher ADL.

As indicated in Table 3, the results showed that living in the urban area, having both burst and compression fracture, higher HRQoL and ADL scores were significantly associated with higher IADL scores. Patients with ASIA-B, however, were significantly associated with a lower IADL score.

## 4. Discussion

This study examined HRQoL, ADL, and IADL among patients who experienced complete spinal cord injury and received surgical treatment. This is the first study providing detailed insights with people having SCI in Vietnam using the three health indices, offering evidence for further health interventions and changes in community health strategies.

Low quality of life scores were observed, with the majority of participants interviewed having suffered from serious health problems including both physical and mental health issues. In our current research, the EQ-5D index among participants was appreciably lower than the general population index (mean = 0.91) [29], suggesting that spinal cord injury is responsible for a significant decrease in HRQOL.

Previous evidence has determined factors that affected HRQoL scores were age, gender, occupation, and living area [30]. In this study, we found that age, marital status, occupational, causes of injuries, ASIA, and IADL were statistically associated with HRQoL. Age is an individual characteristic that cannot be changed. With increasing age, there is a loss of energy and it becomes more difficult to adjust to changes in life [31]. Elderly people were related to a lower score of HRQoL among SCI patients [32,33]. However, in this study, our finding was slightly different in which increased age was correlated with higher HRQOL in the multivariate model. This could be explained by the time of assessment since the SCI injuries have occurred. While previous studies mainly measured quality of life among patients at the time of injury or hospitalization, our study interviewed patients who were treated successfully by surgical approach and came back to hospital for follow-up examination (with an average time of 18.5 ± 5.2 months after surgery). Elderly patients would have the adaptation to the new condition and their self-rated quality of life would be higher. There was no significant difference between the quality of life and gender in this study. Some previous studies also did not reveal any significant differences in HRQoL between men and women in the patient groups [34,35] which is similar to our finding in this study. However, another study showed that women have more health problems than men do [30] and the women also seek more self-medication or health services. Having health insurance, occupation, traffic accidents, ASIA-A were correlated with lower HRQoL. Previous evidence suggested that the occupation of a patient was significantly correlated with global HRQoL injury [36] and the HRQoL scores were significantly lower in unemployed patients [32,37]. Additionally, the HRQoL index is highest in ASIA-D while lowest in ASIA-A. This can be explained the more severe the injury, the lower the quality of life. A better understanding of the degree of impairment will be an important component of efforts to improve the quality of life. Besides, we also did not find a significant difference between quality-of-life scores and type of fracture (with *p* > 0.05).

Besides the HRQOL, the disabilities in activities of daily living were also of physician’s interest. ADL and IADL reflect the ability to function independently and could be the predictor of a patient’s need for support after the surgery. Moreover, identifying factors associated with these disabilities would be useful for clinical practice during treatment. In this study, we found that ADL was significantly associated with gender, occupation, having health insurance, and type of fracture while IADL was significantly correlated with living area, type of fracture, and ASIA. This is in accordance with previous studies which reported that unemployed participants were reported to have the lowest ADL and IADL scores [38,39]. A significant relationship was found between education and ADL/IADL scores in previous evidence [40,41]. People with higher education levels probably have a wider range of abilities, life skills, and participate in more health care activities compared to other people. These capabilities help them to regain independence after trauma. In this study, mean ADL scores were also highest in post-graduate even though there was no statistically significant. In the IADL model, living in urban areas was significantly correlated with higher IADL scores. Our finding is similar to previous studies which showed that residents in urban areas had better outcomes such as quality of life and ADL scores than that of rural areas [39,42]. This can be explained by the number of hospitals that are disproportionally distributed among regions and mainly in urban areas. The problem may indicate that facilities and health workers are not rationally distributed among hospitals, a lack of expertise health workers at the grassroots [43]. Besides, people in rural areas are under much lower cost pressure for health care than those in urban areas [44]. ASIA-B is also associated with lower scores in our study. 

Our study also found that the HRQoL, ADL, and IADL have the interaction between them. In the multivariate Tobit regression model, the increased IADL score was associated with a higher HRQoL (Coef: 0.12; 95% CI: 0.11, 0.14) and higher ADL score (Coef: 1.08, 95%CI: 0.89, 1.27). Meanwhile, increased HRQoL and ADL scores also correlated with a higher IA score. This can be explained according to calculating all these indices’ scores, the better health conditions patients had, the higher points they owned. This also suggested that these three indices were associated with not only patients’ characteristics but also correlated with each other indices and they should be considered in future interventions. 

Improving HRQoL has become the main goal during the rehabilitation process. ADL is a basic activity of daily living, while IADL is an instrumental activity of daily living. Both are very valuable in the evaluation during the rehabilitation of SCI patients. So, identification of factors that influence HRQoL and ADL, IADL is necessary. Some key points should be highlighted in this research. The discomfort outcome during clinical treatment—a factor that directly affects the quality of life should be noticed and treated effectively in the recovery process. Flexion, extension, compression, shear, and rotation are the primary external forces that can be applied to the cervical spine [45]. Pain management for SCI patients is essential as it may reduce morbidity, psychological well-being, and improve long-term effects. Moreover, applying appropriate and widely accepted surgical management such as the anterior approach [46] could also improve surgical outcomes. For cervical vertebra surgery, the investigation and application of novel techniques should be considered. Recently, the anatomical entity named Y-shaped trabecular structure of the odontoid [47] process has been reported which has interesting implications for novel surgical techniques. Furthermore, these participants, especially patients in group unemployment and living in a rural area, had many health problems and exhibited low quality of life scores points to the crucial role of healthcare services which assist them in rehabilitation and provide mental health counseling services to help them improve their functional capacity and reintegrate into society. People experiencing certain types of lesions such as burst fracture and/or compression fracture cervical vertebrae, or neither except for a damaged spinal cord, should be provided special attention and support from responsible people quickly because of the severity of having a lower quality of life scores. 

Nevertheless, this investigation acknowledges some limitations. First, this is a cross-sectional study design instead of follow-up research, all indicators are assessed at one time only after the surgery, therefore these figures do not reflect the disparity of life’s quality before and after the surgical procedure. Besides, though we collected all patients treated in the central surgical hospital from June 2018 to June 2019, there were only 88 people satisfied with the inclusion criteria. The limitations of the convenience sampling technique could have restricted the generalizability of the results for the Vietnamese population. Secondly, the self-report of health status can be considered as a major limitation. The accuracy of self-report may be affected by social desirability, especially in sensitive issues such as depression [48]. Awareness, comprehension, and recall bias are other significant concerns of self-reporting [49]. Additionally, we did not collect information about the level of injury and surgical technique, which leads to a deficiency in the assessment of factors related to the patient’s quality of life. Finally, despite our efforts to avoid specialized terms and complex technical tasks in applied questionnaires, missed classifications still have occurred, similar to other self-reported health surveys [50,51]. Further studies that examine the preference weights for HRQoL, ADL, IADL indices are necessary to assess the population’s quality of life more accurately and can be considered as evidence to compare with our research results.

## 5. Conclusions

In summary, low scores for quality of life were observed among patients suffering from SCIs. Sociodemographic factors and directly related injury factors had certain influences on patients’ quality of life and there is a covariate relationship among the three indices. The disability outcome during clinical treatment should be noticed and treated effectively in the recovery process. Findings from our research can be used as specific evidence for decision-makers to adjust the health policies and healthcare strategies among people having SCIs. Likewise, these results can be used as reference figures for health departments to evaluate the difference in quality of life between SCI patients and other groups of population in Vietnam.

## Figures and Tables

**Table 1:** Characteristics of participants.

Characteristics	Number (*N*)	Proportion (%)
Age	Mean = 47.7 ± 14.6	
<30	11	12.5
30–44	25	28.4
45–59	31	35.2
60+	21	23.9
Gender		
Male	77	87.5
Female	11	12.5
Occupation/job		
White Collar	12	13.6
Blue Collar	9	10.2
Freelancer	10	11.4
Student	2	2.3
Retirement	12	13.6
Unemployment	43	48.9
Educational level		
Primary school or less	16	18.2
Secondary school	26	29.6
High school	28	31.8
College or Higher education	18	20.7
Marital status		
Single/widows/widowers	9	10.2
Married	79	89.8
Living area		
Urban	15	17.1
Rural	73	82.9
Health Insurance Coverage		
Insured	70	79.6
Uninsured	18	20.4
Cause of injuries		
Traffic	45	51.1
Occupational	17	19.3
Home and leisure	26	29.6
Type of fracture		
Burst	27	30.7
Compression	28	31.8
Both burst and compression	13	14.8
Neither burst nor compression	20	22.7
ASIA		
A	20	22.7
B	21	23.9
C	22	25.0
D	25	28.4
Length of stay (days)		Mean = 12.91 ± 5.86
Time from surgery to interview (months)		Mean = 18.5 ± 5.2

**Table 2:** The mean and standard deviation of the HRQoL, ADL, IADL index by patients’ characteristics.

Characteristics	HRQoL	ADL	IADL
Age			
<30	0.65 ± 0.46	4.91 ± 2.07	6.45 ± 2.77
30–44	0.46 ± 0.42	3.64 ± 2.38	5.56 ± 2.74
45–59	0.50 ± 0.50	4.32 ± 2.45	5.90 ± 3.98
60+	0.46 ± 0.49	3.62 ± 2.52	5.00 ± 3.30
Gender			
Male	0.50 ± 0.46	4.01 ± 2.40	5.56 ± 2.96
Female	0.52 ± 0.51	4.18 ± 2.60	6.36 ± 2.94
Health Insurance Coverage			
Insured	0.47 ± 0.47	3.96 ± 2.44	5.69 ± 2.94
Uninsured	0.59 ± 0.44	4.33 ± 2.33	5.56 ± 3.09
Occupation before the accident			
White Collar	0.74 ± 0.26	5.43 ± 5.55	7.19 ± 2.07
Blue Collar	0.44 ± 0.51	3.50 ± 2.61	5.28 ± 3.13
Freelancer	0.48 ± 0.42	4.15 ± 2.22	5.50 ± 2.76
Student	0.97 ± 0.05 *	6.00 ± 0.00 *	8.00 ± 0.00
Retirement	0.22 ± 0.60	2.75 ± 2.49	4.25 ± 3.62
Occupation after the accident			
White Collar	0.84 ± 0.15	5.67 ± 1.15	7.58 ± 1.44
Blue Collar	0.84 ± 0.26	5.56 ± 1.33	7.44 ± 1.67
Freelancer	0.71 ± 0.39	5.50 ± 1.68	7.20 ± 1.62
Student	0.97 ± 0.05 ***	6.00 ± 0.00 ***	8.00 ± 0.00 ***
Retirement	0.36 ± 0.54	3.83 ± 2.55	5.42 ± 3.37
Unemployment	0.30 ± 0.44 ***	2.88 ± 2.40 ***	4.35 ± 3.02 ***
Educational level			
Primary school or less	0.39 ± 0.51	3.19 ± 2.64	5.06 ± 3.19
Secondary school	0.53 ± 0.43	4.15 ± 2.31	5.69 ± 2.99
High school	0.46 ± 0.47	4.18 ± 2.40	5.68 ± 2.97
College or Higher education	0.61 ± 0.48	4.39 ± 2.38	6.11 ± 2.83
Marital status			
Single/widows/widowers	0.59 ± 0.52	4.33 ± 2.24	5.56 ± 3.24
Married	0.49 ± 0.46	4.00 ± 2.44	5.67 ± 2.94
Living area			
Urban	0.63 ± 0.50 *	4.27 ± 2.55	6.07 ± 2.91
Rural	0.47 ± 0.46	3.99 ± 2.39	5.58 ± 2.98
Cause of injuries			
Traffic	0.48 ± 0.45	3.98 ± 2.36	5.53 ± 2.98
Occupational	0.49 ± 0.51	4.35 ± 2.42	6.06 ± 2.77
Home and leisure	0.54 ± 0.47	3.92 ± 2.56	5.62 ± 3.11
Type of fracture			
Burst	0.52 ± 0.47	4.26 ± 2.43	5.59 ± 3.34
Compression	0.59 ± 0.40 *	4.25 ± 2.32	5.96 ± 2.80
Both burst and compression	0.15 ± 0.48	2.54 ± 2.50	4.38 ± 2.72
Neither burst nor compression	0.57 ± 0.47	4.4 ± 2.26	6.15 ± 2.72
ASIA			
A	0.05 ± 0.39 ***	2.05 ± 0.44 ***	3.20 ± 2.98 ***
B	0.38 ± 0.43	3.24 ± 2.36	4.71 ± 2.81
C	0.68 ± 0.34	4.82 ± 1.87	6.59 ± 2.36
D	0.79 ± 0.34 ***	5.60 ± 1.29 ***	7.60 ± 1.61 ***

* *p* < 0.05, *** *p* < 0.001.

**Table 3:** Multivariate Tobit regression analysis for HRQOL, ADL, IADL index scores.

Characteristics	HRQol		ADL		IADL	
	aCoef.	95%CI	aCoef.	95%CI	aCoef.	95%CI
Age	0.003 *	0.00; 0.07				
Female (vs. male)			−2.53 *	−4.47; −0.59		
Health Insurance (Yes vs. No)	−0.11 *	−0.21; −0.006	−1.63 **	−2.83; −0.43	0.91	−0.07; 1.90
Occupation after the accident (vs. White Collar)						
Blue-collar	0.006	−0.17; 0.18	−1.27	−4.31; 1.75		
Freelancer	−0.07	−0.24; 0.09	−1.61	−4.61; 1.38		
Student	0.24	−0.10; 0.59	2.60	−575.9; 581.2		
Retirement	−0.29 ***	−0.46; −0.12	−2.74 *	−5.33; −0.15		
Unemployment	−0.06	−0.20; 0.08	−4.78 **	−7.72; −1.84		
Educational level (vs. Primary school or less)						
Secondary school			−0.11	−1.38; 1.17		
High school			0.84	−0.31; 2.00		
College or Higher education			−1.13	−2.97; 0.69		
Marital status (vs. Single/widows/widowers)	0.16 *	0.01; 0.31				
Urban area (vs. rural area)	0.10	−0.02; 0.22			1.37 *	0.04; 2.68
Causes of injuries (vs. home and leisure)						
Occupational	−0.03	−0.15; 0.10	1.17	−0.17; 2.53		
Traffic	−0.10 *	−0.20; −0.0007	0.23	−0.82; 1.29		
Types of frature (vs. neither burst fracture nor compression fracture)						
Compression	0.09	−0.04; 0.22	2.03 **	0.64; 3.41	−0.04	−1.23; 1.15
Burst	0.10	−0.08; 0.22	1.46 *	0.09; 2.83	0.16	−0.95; 1.28
Burst and compression	−0.04	−0.18; 0.10	0.58	−0.84; 2.01	1.58 *	0.22; 2.95
ASIA (vs. D)						
A	−0.25 **	−0.41; −0.09			−0.62	−2.26; 1.02
B	−0.05	−0.18; 0.08			−1.48 *	−2.93; −0.02
C	0.01	−0.11; 0.12			−1.39	−2.89; 0.11
HRQoL ^a^					5.05 ***	3.34; 6.77
ADL ^a^					0.79 ***	0.52; 1.07
IADL ^b^	0.12 ***	0.11; 0.14	1.08 ***	0.89; 1.27		

* *p* < 0.05, ** *p* <0.01, *** *p* < 0.001; aCoef, adjusted Coefficient; ^a^ included in IADL model only; ^b^ included in HRQoL and ADL models.

## Data Availability

The data that support the findings of this study are available from the corresponding author upon reasonable request.
